# Altering an Artificial Gagpolnef Polyprotein and Mode of ENV Co-Administration Affects the Immunogenicity of a Clade C HIV DNA Vaccine

**DOI:** 10.1371/journal.pone.0034723

**Published:** 2012-04-11

**Authors:** Katharina Böckl, Jens Wild, Simon Bredl, Kathrin Kindsmüller, Josef Köstler, Ralf Wagner

**Affiliations:** 1 Institute of Medical Microbiology, University of Regensburg, Regensburg, Germany; 2 Geneart AG/Life Technologies, Regensburg, Germany; Istituto Superiore di Sanità, Italy

## Abstract

HIV-1 candidate vaccines expressing an artificial polyprotein comprising Gag, Pol and Nef (GPN) and a secreted envelope protein (Env) were shown in recent Phase I/II clinical trials to induce high levels of polyfunctional T cell responses; however, Env-specific responses clearly exceeded those against Gag. Here, we assess the impact of the GPN immunogen design and variations in the formulation and vaccination regimen of a combined GPN/Env DNA vaccine on the T cell responses against the various HIV proteins. Subtle modifications were introduced into the GPN gene to increase Gag expression, modify the expression ratio of Gag to PolNef and support budding of virus-like particles. I.m. administration of the various DNA constructs into BALB/c mice resulted in an up to 10-fold increase in Gag- and Pol-specific IFNγ^+^ CD8^+^ T cells compared to GPN. Co-administering Env with Gag or GPN derivatives largely abrogated Gag-specific responses. Alterations in the molar ratio of the DNA vaccines and spatially or temporally separated administration induced more balanced T cell responses. Whereas forced co-expression of Gag and Env from one plasmid induced predominantly Env-specific T cells responses, deletion of the only H-2^d^ T cell epitope in Env allowed increased levels of Gag-specific T cells, suggesting competition at an epitope level. Our data demonstrate that the biochemical properties of an artificial polyprotein clearly influence the levels of antigen-specific T cells, and variations in formulation and schedule can overcome competition for the induction of these responses. These results are guiding the design of ongoing pre-clinical and clinical trials.

## Introduction

In view of the 2.6 million new HIV infections in 2009 alone, developing a prophylactic and therapeutic human immunodeficiency virus type-1 (HIV-1) vaccine remains one of the most desirable objectives of research aimed at controlling the current AIDS epidemic.

Evidence from natural infection studies with long-term non-progressors (LTNPs) suggests that substantial CD8^+^ T cell responses to Gag are correlated with better control of HIV infection [1]–[5]. Hence a variety of T cell based vaccine candidates are under evaluation.

One recent reversal in HIV-1 vaccine development was the failure of a clinical efficacy study, the STEP trial [6]–[8], using a homologous prime∶boost regimen of a 1∶1∶1 mixture of three separate replication-defective Ad5 vectors, expressing HIV-1 Gag (CAM-1 strain), Pol (IIIB strain) and Nef (JR-FL strain). Not only did the vaccine fail to protect Ad5-seronegative individuals against infection with HIV-1, or reduce viral load, but further analysis also suggested that the vaccine might have actually increased susceptibility to infection in vaccinees with prior immunity to adenoviruses. A variety of hypotheses were discussed to explain the increased susceptibility to HIV infection, but the mechanism(s) responsible for the enhanced sensitivity in vaccine recipients with high Ad5-NAb titers is still elusive.

In a subsequent Phase III clinical trial, RV-144, also called Thai-Trial, HIV infections were prevented with a 31% reduced infection rate among vaccinees compared to the placebo group [9]. Treatment involved administering the poxvirus ALVAC-HIV and a recombinant gp120 subunit vaccine (AIDSVAX B/E) in a heterologous prime∶boost regime. Although the vaccine showed no effect on the levels of viremia or CD4^+^ T cell counts in subjects infected during the study, this clinical trial demonstrated for the first time that an HIV vaccine is able to prevent HIV infection, and that recombinant poxvirus vectors are a safe and effective tool for vaccination in humans.

Several clinical studies, including EV02 and EV03, have since confirmed in various prime∶boost regimens the safety and immunogenicity of poxviral vectors [10]–[12]. In the EV02 trial, some HIV negative volunteers received two injections of an equimolar mixture of two plasmids encoding an artificial GagPolNef (DNA-C) polyprotein and the external glycoprotein gp120 [13], followed by two booster immunizations with a poxviral (NYVAC) vectored vaccine co-expressing the identical set of immunogens (NYVAC-C; New York Vaccinia Virus). The other group received only two NYVAC-C immunizations. Both the artificial 160 kDa cytoplasmic and nonglycosylated GagPolNef antigen as well as the secreted form of the viral envelope protein gp120 were encoded by RNA and codon optimized genes derived from a previously described clade C virus (CN54) representing the most prevalent HIV-1 strain circulating throughout China [14].

Whereas the homologous prime∶boost with NYVAC-C induced only marginal antigen-specific T cell responses [15], the heterologous DNA-C∶NYVAC-C prime∶boost regimen resulted in durable (at week 72 in 70% of vaccinees), broad (average number of 4.2 epitopes per responder) and polyfunctional (characterized by the production of multiple cytokines, e.g. IFNγ and IL-2) T cell responses, which consisted of both CD4^+^ and CD8^+^ T cells. Notably, these detected T cell responses mimic T cell profiles seen in LTNPs [10]. Furthermore, a modified DNA-C∶NYVAC-C prime∶boost regime in an additional Phase II clinical trial (EV03) induced the homing of these potentially protective T cells into the gut [12], which represents the most prominent portal of entry for HIV.

Despite these encouraging results, EV02 also demonstrated that Env-specific T cell responses dominated over Gag, Pol, or Nef specific responses; at week 26, the median of Env-specific IFNγ secreting T cells measured by ELISpot was 299 SFU/10^6^ cells, whereas Gag, Pol, and Nef responses reached only about 100 SFU and were no longer detectable at week 48. The same tendencies were basically observed in pre-clinical trials with both mice and rhesus macaques [13], [16].

Therefore, the aim of this study was to improve T cell responses directed against the highly conserved Gag and Pol component by improving the Gag-Pol-Nef immunogen design, and achieve more balanced T cell responses without affecting the well-induced gp120-specific responses.

## Materials and Methods

### Ethics statement

All experiments were conducted in accordance with the legal requirements of local and national authorities. According to the German Animal Welfare Act, article 10a, the presented mouse study did not require the approval by the local authorities, since exclusively standardized immunization protocols were used. Thus, notification of the animal studies to the competent authority (Regierung der Oberpfalz, Bavaria, Germany) two weeks before beginning was sufficient. All animal studies were supervised by an animal welfare officer.

### Plasmid constructs

The RNA- and codon-optimized GagPolNef and Env gene constructs were designed by Geneart (Life Technologies) (Regensburg, Germany) as described previously [13]. All gene constructs were finally cloned in a pcDNA3.1 vector under a CMV promoter. The original Clade C/B′ GagPolNef (GPN) and Env (gp120) sequences were designed based on sequence information derived from a 97CN54 provirus clone [14] (Accession Nr. AX149647). The RNA- and codon-optimized CN54 Env construct comprises 1500 nucleotides encoding an artificial signal peptide (MDRAKLLLLLLLLLLPQAQA) [17] followed by gp120 CN54 (nt 5673–7109).

The reference construct 97CN54 GPN, henceforth called ^ΔM^GPN, was described recently [10], [13], [16]. Briefly, it encodes a ∼160 kDa non-glycosylated artificial polyprotein comprising (i) a myristoylation deficient group-specific antigen fused to (ii) the 5′ part of *pol* including the inactivated viral protease, followed by (iii) a scrambled Nef variant (5′ end linked to 3′ end) replacing the active site of the reverse transcriptase, (iv) the 3′ pol reading frame lacking the integrase gene, extended by (v) the 3′ end of the scrambled Nef gene, followed by (vi) a sequence stretch encoding the active site of the reverse transcriptase.

To restore the competence to form virus-like particles (VLP, budding competence) the myristoylation signal (A2G) was replaced, but this did not result in the formation of particles. Aligning the sequences of 97CN54 with a replication competent virus isolate (97CN001) from the same patient revealed seven amino acid exchanges in regions known to be involved in the aggregation of Gag proteins. Additional substitution of these amino acids (P66S, L85P, K98R, D309G, R439G, G447R, and G461E) finally allowed VLP formation. Subsequently, this codon-optimized Gag sequence was cloned into the *Kpn*I and *EcoR*I restriction sites of the reference construct. To restore the HIV ribosomal frameshift, the frameshift sequence was synthesized by Geneart (Life Technologies) and subsequently cloned into the *Age*I and *BsaB*I restriction sites of the reference construct. All new GPN derivatives, i.e. (i) the construct ^M^GPN encoding the 160 kDa read-through protein with the myristoylation signal and seven substituted amino acids within Gag; (ii) ^ΔM^G^FS^PN, containing the original frameshift, and (iii) ^M^G^FS^PN, containing all the modifications to restore budding competence and the frameshift sequence, were cloned via *Kpn*I and *Xho*I into the expression vector pcDNA3.1(+) (Invitrogen). Furthermore, the modified Gag sequence was cloned into the *Kpn*I and *EcoR*I restriction sites of the expression vector pcDNA3.1(+) (Invitrogen) resulting in the construct ^M^Gag.

97CN54 PolNef was obtained from the original 97CN54 GPN construct via PCR and was subsequently cloned into the *Kpn*I and *Xho*I restriction sites of the expression vector pcDNA3.1(+) (Invitrogen) resulting in the construct PN.

Plasmid pQL11 (kind gift from Tim-Henrik Bruun, Regensburg) was used to amplify a sequence encoding the human EGFP (Clontech), the 18 amino acid TaV2a peptide EGRGSLLTCGDVEENPGP (called 2a) [18] and the ccdB (synthesized by Geneart (Life Technologies)) by PCR. The resulting product was subcloned between the *Kpn*I and *Xho*I sites of pcDNA3.1(+) (Invitrogen) to generate the vector pcEGFP-2a-ccdB. The 97CN54 Gag and CN54 Env (gp120) were obtained from the original pcDNA3.1(+) vectors via PCR; subsequently, EGFP and ccdB were replaced by Gag and Env upstream and downstream of 2a, respectively. In each case, the upstream open reading frame (ORF) lacked the termination codon, while the downstream ORF is full length.

The pcgp120^ΔV11V^ construct based on the original CN54 Env (gp120) was generated by deleting the sequence of the BALB/c restricted gp120-specific epitope V11V (VPADPNPQEMV) in the gp120 reading frame. Afterwards, the gene was cloned into pcDNA3.1(+) (Invitrogen).

All pDNAs were prepared as supercoiled purified solutions using Qiagen Megaprep Endo-free kits (Qiagen). For in vivo application, pDNAs were resuspended in sterile saline solution.

### Synthetic peptides

All peptides were purchased from BioSynthan and dissolved in DMSO at 10 µg/µl. The Gag p24(CA)-derived 9-mer peptide A9I ‘C-clade’ (AMQILKDTI), the Pol derived 9-mer peptide L9I (LVGPTPVNI) [13] and the gp120 derived 11-mer peptide V11V (VPADPNPQEMV) [13] were used to assess specific CD8^+^ T cell responses.

### Cell lines and transfections

293T cells (human embryonal kidney cells, HEK) (DSMZ, no.: ACC-635) were maintained in Dulbecco's modified Eagle medium (Invitrogen) supplemented with 10% fetal calf serum, 2 mM L-glutamine, 100 IU of penicillin and 100 µg streptomycin per ml. Cells were seeded into 6-well tissue plates and grown overnight to 80% confluency. Transfection in duplicate wells was performed using jetPEI transfection reagent (PeqLab) according to the manufacturer's instructions. Briefly, cells were transfected with 3 µg of plasmid DNA and 6 µl of jetPEI in a total volume of 2 ml Dulbecco's modified Eagle medium (Invitrogen) including 10% fetal calf serum and 2 mM L-glutamine without antibiotics. Following 48 h incubation, supernatants were harvested, pelleted cell lysates collected, and both stored at −20°C. For immunoblotting, supernatants were precipitated by adding 100% TCA in a volume ratio of 1∶10 and subsequently washed with 200 µl of acetone. Cell lysis was performed by incubation with RIPA buffer for 1 h and a subsequent sonification step.

### Immunoblotting

Immunoblotting was performed as described earlier [19]. Gag and GagPolNef were detected using a p24-specific monoclonal mouse antibody (CB-4/1, 1∶1000) and a horseradish peroxidase-conjugated goat anti-mouse immunoglobulin G (Dianova; 1∶10,000 dilution including 3% milk powder). 97CN54 PolNef was detected using a HIV-1 RT-specific monoclonal mouse antibody (clone 5B2B2, HIV molecular immunology database, D. Helland; 1∶400 dilution including 1% milk powder) and a horseradish peroxidase-conjugated goat anti-mouse immunoglobulin G (Dianova; 1∶10,000 dilution including 3% milk powder). Env CN54 was detected using a gp140-specific monoclonal mouse antibody (clone MH23, Mark Hassal NIBSC; 1∶1,000 dilution including 1% milk powder) and a horseradish peroxidase-conjugated goat anti-mouse immunoglobulin G (Dianova; 1∶10,000 dilution including 3% milk powder).

### Vaccination of mice

Female BALB/c mice (Charles River) were immunized at the age of 8 to 12 weeks. Groups of 6 mice were immunized by intramuscular (i.m.) saline injection of 50 µl into both *tibialis anterior* muscles. Equimolar amounts of plasmid DNA were applied (unless indicated otherwise: pc^ΔM^GPN, 80 µg; pc^M^GPN, 80 µg; pc^ΔM^G^FS^PN, 80 µg; pc^M^G^FS^PN, 80 µg; pcGag, 57 µg; pcPN, 68 µg; pcgp120, 57 µg; pc^M^Gag-2a-gp120, 70 µg; pcgp120^ΔV11V^, 57 µg). When administering two plasmid DNAs at the same time, DNA was applied as either a mixture in both legs, or separated preparations in the left or right leg.

### FACS analyses

IFNγ expression of CD8^+^ T cells was detected by intracellular staining followed by FACS analyses. Splenocytes were stimulated with 10 µM peptide in RPMI medium or medium alone as a negative control for 6 h in the presence of Brefeldin A (5 mg/ml). Intracellular staining was performed as described earlier [20]. In total, 3×10^4^ CD8^+^ lymphocytes were analyzed.

Intracellular p24- and gp120-specific staining of transfected 293T cells was performed by incubation of permeabilized cells with fluorochrome-labeled antibodies (anti-p24-antibody, anti-gp120-antibody HGN194) for 25 min. Anti-p24-antibody was purchased as a conjugate with Phycoerythrin (clone KC57-RD1, Beckman Coulter). HGN194 antibody was labeled with ALEXA-488 (Protein Labeling Kit, Invitrogen) according to the manufacturer's instructions. Cells were analyzed by flow cytometry using BD FACS Canto II and DIVA 6.0 software (BD).

## Results

**Figure 1:**
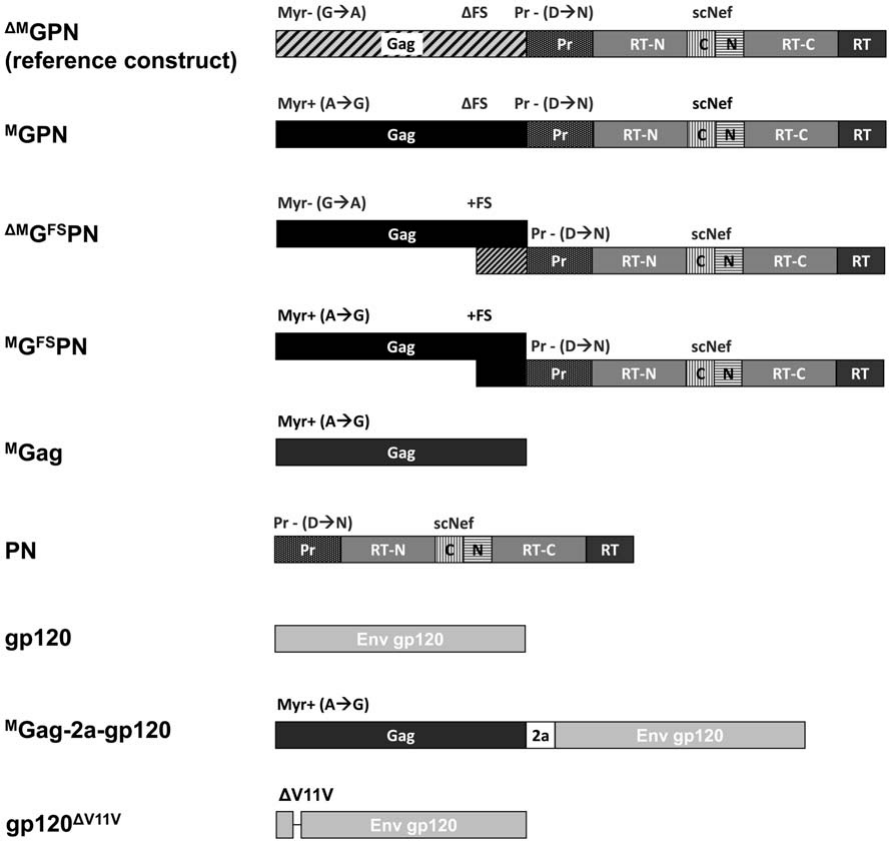
Schematic representation of synthetic expression cassettes derived from gag-pol-nef as well as env (gp120). The reference construct ^ΔM^GPN encodes a 160 kDa read-though protein (ΔFS) comprising the gag-derived domains p17 (matrix), p24 (capsid), p1, p7, p2 and p6* plus the mutated myristoylation signal (Myr- (G→A)); the pol-derived domains protease (Pr), the N- and C-terminal portions of the reverse transcriptase (RT-N, RT-C) plus its active site (RT-AS); a scrambled nef (scNEF); and further modifications for safety [13]. ^M^GPN contains a restored myristoylation signal (Myr+ (A→G)) and additional exchanged sequences of 7 amino acids. To generate ^ΔM^G^FS^PN, the original natural frameshift sequence (+FS) of the CN54 virus isolate was introduced into the ^ΔM^GPN reference construct. ^M^G^FS^PN contains both the myristoylation signal/amino acid exchange modifications and the natural frameshift. ^M^Gag contains the Gag sequence with the myristoylation signal and the amino acid sequence modifications. PN encodes the Pol and Nef domains including all the modifications of these reading frames as in the reference construct. The codon optimized CN54 derived Env gp120 sequence contains an artificial signal peptide (SP) replacing that of native HIV Env. ^M^Gag-2a-gp120 contains the ^M^Gag and gp120 reading frames separated by the 18 amino acid TaV2a peptide from *Thosea asignia* virus. To generate gp120^ΔV11V^ the gp120-specific epitope V11V was deleted in the CN54 Env gp120 sequence. All derivatives are under the control of the CMV promoter.

Since we wanted to improve Gag, (Pol, and Nef)-specific T cell responses, and achieve more balanced T cell responses directed against these antigens without affecting the well-induced gp120-specific responses, we set out to design, generate, and biochemically characterize various modified Gag-Pol-Nef sequences. All modifications were based on the GPN antigen used for immunization in the EV02 clinical trial, which was a fusion protein comprising Gag, Pol and Nef, including some sequence modifications to enhance its safety profile [13], [16]. The main characteristics of that construct are: (i) in-frame fusion of the *polnef* coding sequence to the *gag* gene resulting in a 160 kDa read-through protein, and (ii) substitution of the N-terminal glycine by alanine, which destroyed the myristoylation site, thereby preventing the budding of virus-like particles (see reference construct, Fig. 1).

### Design and generation of immunologically improved GPN constructs

To increase expression of the encoded antigens, and at the same time, allow the budding and release of VLP to support cross presentation and cross priming events, we introduced a series of modifications stepwise into the Gag-Pol-Nef coding sequence. To allowing budding and particle release, we restored the original myristoylation signal and exchanged seven amino acid sequences putatively essential for budding, resulting in the construct ^M^GPN (Fig. 1). Furthermore, the original 97CN54 ribosomal frameshift sequence with the natural slippery site was reintroduced (^ΔM^G^FS^PN and ^M^G^FS^PN), to obtain a predicted Gag∶PolNef expression ratio of between 10∶1 to 20∶1 [21]. In addition, we generated constructs allowing expression of the budding competent Gag (^M^Gag) and PolNef (PN) from separate plasmids (Fig. 1).

The gp120 gene used in the experiments was identical to that already analyzed in our earlier pre-clinical and clinical studies [11], [13].

### Transgene expression of the GPN derivatives and gp120 in 293T cells

**Figure 2:**
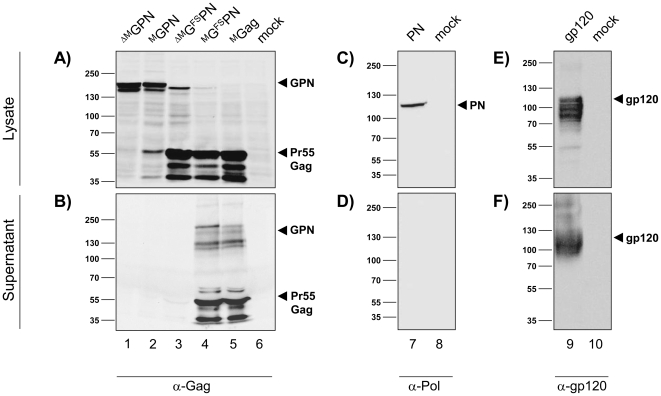
Protein expression from the modified constructs. 293T cells were transiently transfected with the indicated plasmid DNA constructs using jetPEI. Cells and supernatants were harvested after 48 h. Cell lysates, and supernatants processed by TCA/acetone precipitation, were separated by 8.0% SDS-PAGE and analyzed by immunoblotting using p24-specific (Gag) mouse mAb (CB-4/1), HIV-1 RT (Pol)-specific mouse mAb (5B2B2), and gp120-specific mouse mAb (MH23) to specifically detect the corresponding recombinant polypeptides. Molecular weight markers and positions of the specifically detected proteins are indicated. mock: non-transfected 293T cells.

To confirm proper expression of the modified antigens, we transiently transfected human 293T cells with DNA constructs ^ΔM^GPN and ^M^GPN, both of which robustly expressed the 160 kDa GPN polyprotein as detected in cell lysates (Fig. 2A). In addition, ^M^GPN shows a weak band of Pr55Gag, which might result from residual activity of a modified viral protease triggered by membrane targeting and assembly.

Introduction of the natural ribosomal frameshift led to efficient production of Pr55Gag, whereas now only weak signals of the 160 kDa polyprotein GPN were detected, confirming a postulated 10∶1 to 20∶1 ratio of Gag to GagPolNef resulting from the −1 frameshift [21]. Due to the overlapping Gag and Pol reading frame following the frameshift signal, the resulting GPN protein migrates at a slightly lower molecular weight (compared to 160 kDa) than the read-through constructs ^ΔM^GPN and ^M^GPN, where PolNef has been fused to the C-terminus of Gag. Comparable or even slightly increased synthesis of Pr55Gag was observed in all lysates after transfection of ^M^Gag.

In the supernatants, no Gag-specific products were detected in the case of ^ΔM^GPN, ^M^GPN and ^ΔM^G^FS^PN, confirming that both the N-terminal myristoylation, as well as full length 55 kDa Gag expression are required to drive particle formation and release (Fig. 2B). In contrast, Gag-specific products could be detected in the supernatants of cells transfected with ^M^G^FS^PN or ^M^Gag, thus also confirming earlier data demonstrating that RNA and codon optimization overcomes restrictions regarding Gag expression and particle release in murine, monkey as well as human cell lines [19].

As expected, PolNef was only detectable in the cell lysate and not in the supernatant (Fig. 2C, D), indicating that there was no release of PolNef. In contrast, gp120 was readily detected in both the cell lysate and supernatant (specific bands of lower molecular weight result from different glycosylation patterns) (Fig. 2E, F).

### Budding competence and ribosomal frameshift enhance Gag- and Pol-specific CD8^+^ T cell responses

**Figure 3:**
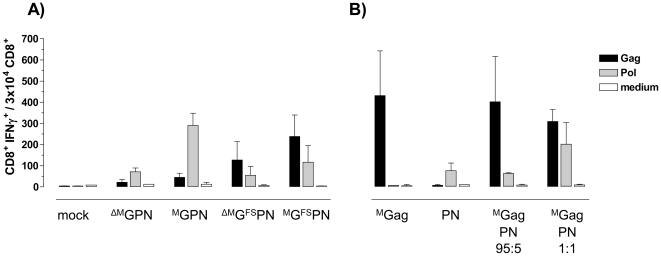
Influence of the modified constructs on Gag- and Pol-specific CD8^+^ T cell immune responses. BALB/c mice (n = 6 per group) were i.m. inoculated with equimolar amounts of the indicated plasmid DNA constructs (80 µg of ^ΔM^GPN, ^M^GPN, ^ΔM^G^FS^PN, ^M^G^FS^PN; 57 µg of ^M^Gag; 68 µg of PN). After 12 days, spleen cells were isolated and tested for specific cellular immune responses by measuring IFNγ production after stimulation with Gag (black) or Pol (grey) specific peptides. IFNγ production was determined by FACS analysis after intracellular staining of IFNγ. Cell culture medium served as a negative control. (A) Influence of budding competence and the functional frameshift on Gag- and Pol-specific CD8^+^ T cell immune responses. (B) Impact of separating the Gag and PolNef reading frames on Gag- and Pol-specific CD8^+^ T cell responses. Data shown are representative of two experiments.

To determine the impact of our modifications on the induction of specific T cell responses, we inoculated BALB/c mice (n = 6 per group) i.m. with 80 µg of ^ΔM^GPN, ^M^GPN, ^ΔM^G^FS^PN or ^M^G^FS^PN. After 12 days, isolated splenocytes were stimulated in vitro with a known Gag- or Pol-specific peptide, as well as medium alone as a negative control. Subsequently, we determined the frequency of CD8^+^ IFN-γ^+^ T cells by FACS analysis. Non-immunized BALB/c mice served as the negative control group (Fig. 3A).

The myristoylation deficient, budding-incompetent reference construct ^ΔM^GPN expressing a 160 kDa read-through polypeptide induced only weak Pol-specific T cell responses, and Gag-specific responses were scarcely above the background level, affirming former experiments [13]. Restoring the myristoylation site slightly increased the number of Gag-specific CD8^+^ T cells by a factor of 2. Interestingly, Pol-specific T cell responses were enhanced by a factor of 3, even though the in vitro expression pattern did not differ compared to ^ΔM^GPN and no particle release was observed.

Introduction of the original frameshift (^M^G^FS^PN) clearly improved Gag-specific T cell responses, however, at the cost of the number of Pol-specific CD8^+^ T cells. This observation correlates with the changed Gag∶GagPolNef ratio already detected above in Western blot analysis. As already seen for ^M^GPN and ^ΔM^GPN, also in the case of ^M^G^FS^PN and ^ΔM^G^FS^PN the recovery of budding competence enhanced both Gag- and Pol-specific CD8^+^ T cell responses, but now responses were reversed, with Pol responses poorer than those to Gag. Remarkably, using a budding competent Gag-only construct (^M^Gag; Fig. 3B), specific T cell responses could be enhanced 10-fold in comparison to the reference construct ^ΔM^GPN (233±109 compared to 20.5±14.8).

### Balancing Gag- and Pol-specific immune responses with multivalent Gag and PolNef vaccine strategies

Besides the above tested constructs encoding Gag, Pol and Nef linked in an open reading frame, BALB/c mice (n = 6 per group) were also inoculated with ^M^Gag or PolNef (PN) on separate plasmids. Furthermore, both constructs were mixed at equimolar amounts or at a Gag∶Pol ratio of 20∶1 to approximately mimic the effect of the viral ribosomal frameshift.

As expected, the mixture of ^M^Gag and PN in a 20∶1 ratio induced the highest Gag-specific but lower Pol-specific T cell responses (Fig. 3B), thereby reflecting the tendency already seen in the frameshift vs. the read-through construct (Fig. 3A). Immunization with a 1∶1 mixture of ^M^Gag and PN resulted in a more balanced, but nevertheless efficient Gag- and Pol-specific immune responses for both antigens. Administration of ^M^Gag and PolNef (PN) alone led to the induction of Gag- and Pol-specific T cell responses at levels comparable to the 1∶1 mixture immunization.

### Co-administration of gp120 reduces Gag- and Pol-specific CD8^+^ T cell responses

**Figure 4:**
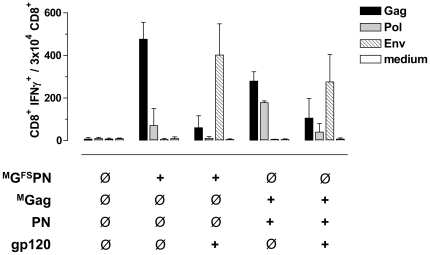
Effect of co-immunization with Gag-Pol-Nef and gp120 on Gag- and Pol-specific CD8^+^ T cell responses. BALB/c mice (n = 6 per group) were i.m. inoculated (+) with ^M^G^FS^PN; or equimolar mixtures of ^M^G^FS^PN and gp120; ^M^Gag and PN; or ^M^Gag, PN, and gp120. After 12 days, spleen cells were isolated and tested for specific cellular immune responses by measuring IFNγ production after stimulation with Gag (black), Pol (grey) or Env (hatched) specific peptides as described in Fig. 3. Cell culture medium served as a negative control. Data shown are representative of two experiments.

Next, to evaluate a possible negative impact of Env DNA-immunization on the outcome of Gag- and Pol-specific T cell responses following co-administration of GPN and gp120, we immunized BALB/c mice (n = 6 per group) with ^M^G^FS^PN or an equimolar mixture (1∶1) of ^M^Gag and PN with or without equimolar amounts of gp120 (1∶1∶1) (Fig. 4). Interestingly, co-administration of gp120 dramatically reduced Gag- and Pol-specific CD8^+^ T cell responses, in the case of ^M^G^FS^PN about 7-fold for both Gag and Pol (Gag: 476±79 to 60.5±56; Pol: 70.5±79 to 10±8.5) and in the case of the ^M^Gag∶PN 1∶1 mixture by at least a factor of 2.6 for Gag (279±44 to 105.5±91) and 4.5 for Pol (178±8.5 to 39.5±40). In contrast, Env responses were not affected, neither by co-immunization of gp120 with ^M^G^FS^PN nor with PN or ^M^Gag.

**Figure 5:**
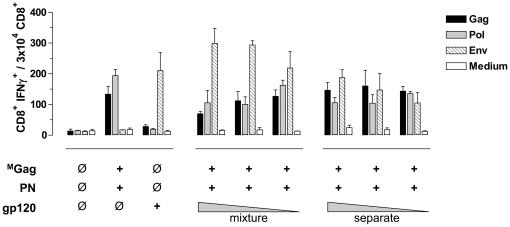
Spatially separating Gag-Pol-Nef and gp120 alters Gag- and Pol-specific CD8^+^ T cell responses. BALB/c mice (n = 6 per group) were i.m. inoculated (+) in both legs with an equimolar mixture of ^M^Gag and PN; gp120 alone, or a mixture of ^M^Gag, PN and gp120, where the amount of gp120 was titrated (mixture: 57, 30, 10 µg). Further groups received plasmid DNAs spatially separated as a mixture of ^M^Gag and PN in the right leg and titrated amounts (separate: 57, 30, 10 µg) of gp120 in the left leg. After 12 days, spleen cells were isolated and tested for specific cellular immune responses by measuring IFNγ production after stimulation with Gag (black), Pol (grey) or Env (hatched) specific peptides. IFNγ production was determined by FACS analysis after intracellular staining of IFNγ as described in Fig. 3. Cell culture medium served as a negative control. Data shown are representative of two experiments.

To further evaluate whether the rate of Gag- and Pol-specific immune response reduction depends on the amount of inoculated gp120, BALB/c mice (n = 6 per group) received an equimolar mixture of ^M^Gag and PN (1∶1) as well as decreasing amounts of gp120 (57, 30, and 10 µg) (Fig. 5, mixture). Correlating with declining amounts of gp120 encoding plasmid DNA, we detected decreasing numbers of gp120-specific CD8^+^ T cells. In contrast, Gag- and Pol-specific immune responses increased gradually as the gp120 amount declined, and finally reached CD8^+^ T cell numbers comparable to those detected after inoculation of ^M^Gag and PN without gp120. This clearly shows that the level of Gag- and Pol-specific T cell responses inversely correlates with the amount of co-administered gp120 plasmid DNA.

### Spatial and temporal separation of Gag-Pol-Nef and gp120 overcome the suppressive effects of Env on Gag- and Pol-specific CD8^+^ T cell responses

Subsequently, we evaluated to what extent the suppressive effect of gp120 co-administration could be overcome by spatial or temporal separation of the Gag, PN, and gp120 encoding plasmids. BALB/c mice (n = 6 per group) were immunized with a mixture of ^M^Gag and PN in the *tibialis anterior muscle* of the left leg, and different amounts of gp120 (57, 30, or 10 µg) in the right leg (Fig. 5, separate). In contrast to the gp120-specific T cell responses, which decreased as expected, the number of Gag- and Pol-specific CD8^+^ T cells remained constantly at a level comparable to the group receiving the mixture of only ^M^Gag and PN.

**Figure 6:**
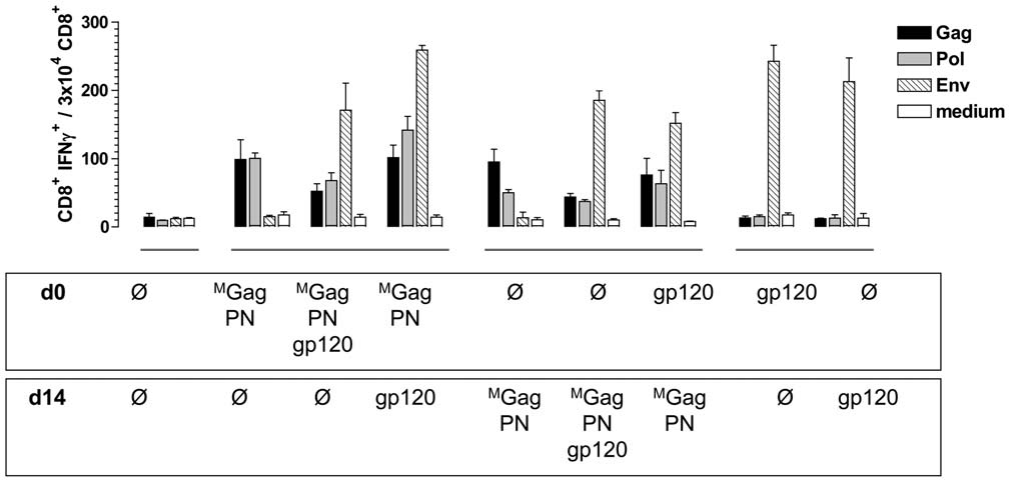
Effect temporal separation of Gag-Pol-Nef and gp120 administration on Gag- and Pol-specific CD8^+^ T cell responses. BALB/c mice (n = 6 per group) were i.m. inoculated at day 0 with a mixture of ^M^Gag and PN; ^M^Gag, PN, and gp120; or ^M^Gag and PN, followed by a booster immunization with gp120 two weeks later (d 14); or vice versa, a mixture of ^M^Gag and PN, or ^M^Gag, PN and gp120 at day 14; or primed at day 0 with gp120 followed by a booster immunization with ^M^Gag and PN at day 14. At day 29, spleen cells were isolated and tested for specific cellular immune responses by measuring IFNγ production after stimulation with Gag (black), Pol (grey) or Env (hatched) specific peptides. IFNγ production was determined by FACS analysis after intracellular staining of IFNγ. Cell culture medium served as a negative control. Data shown are representative of two experiments.

This result prompted us to analyze the impact of temporally separated administration of the plasmid DNAs. Here, BALB/c mice (n = 6 per group) were primed with ^M^Gag and PN at day 0 and boosted 14 days later with gp120, or *vice versa*, primed with gp120 at day 0 and boosted 2 weeks later with ^M^Gag and PN (Fig. 6). In addition, BALB/c mice received a mixture of ^M^Gag and PN, or a mixture of ^M^Gag, PN, and gp120 on day 0 or day 14. As controls, BALB/c mice were inoculated with gp120 at day 0 or 14, and non-immunized mice served as a negative control. Whereas co-administration of gp120 at equimolar amounts, as before, impaired the amount of Gag- and Pol-specific CD8^+^ T cells, temporally separated immunization with ^M^Gag/PN and gp120 led to little reduction in the specific immune responses, generating the same amount of Gag- and Pol-specific T cells as the mixture of ^M^Gag and PN alone.

These experiments clearly demonstrate that, in contrast to applying a mixture of all three plasmid DNAs, spatially separated (^M^Gag and PN in the left leg, gp120 in the right) or temporally separated (day 0 and day 14) administration of the plasmids overcomes the suppressive effect of gp120 on Gag- and Pol-specific CD8^+^ T cell responses.

### Linked Gag-Env co-expression confirms suppressive effects of Env on the induction of Gag-specific T cells

**Figure 7:**
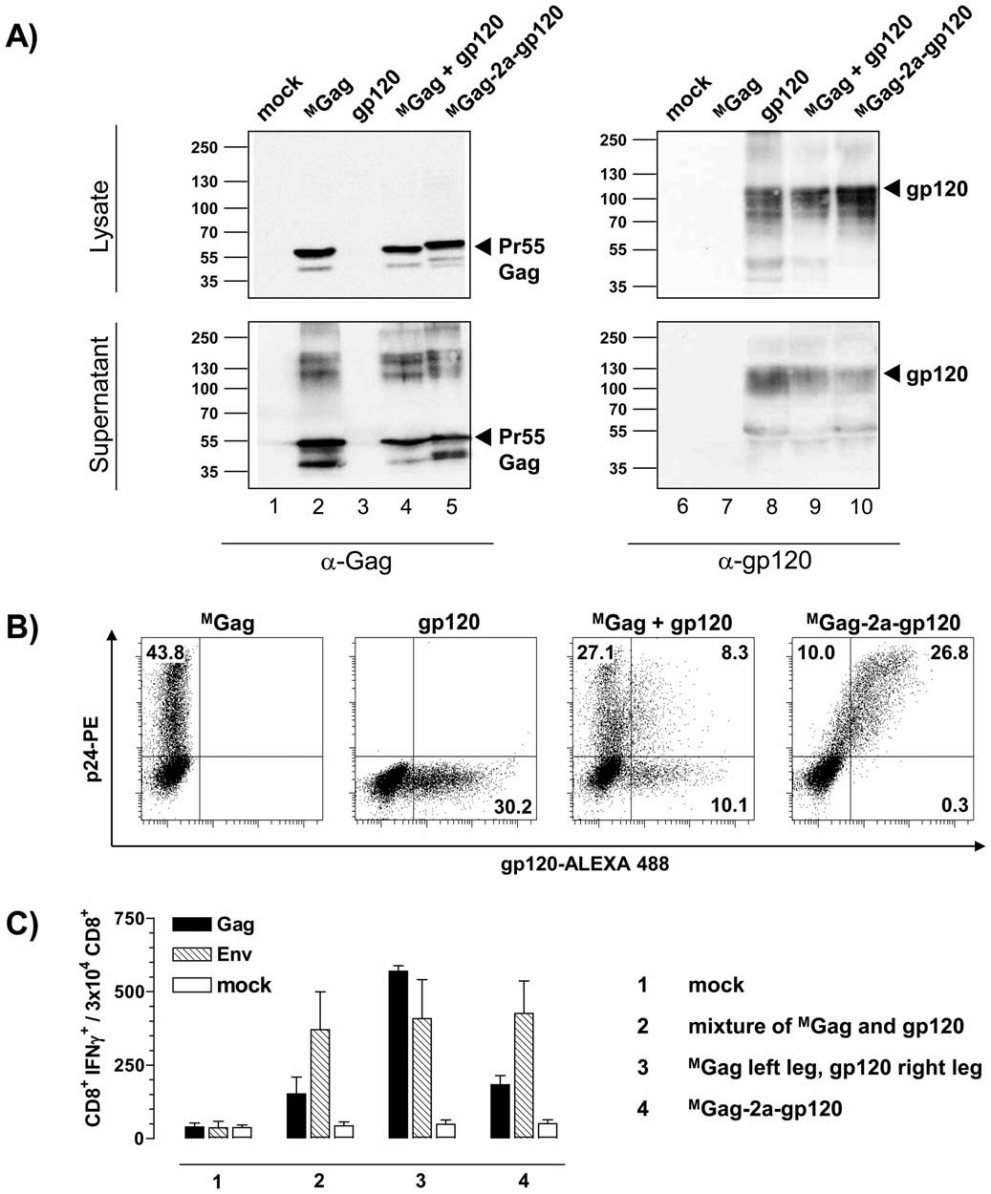
Expression of Gag and Env in co-transfected 293T cells and the effect of administrating Gag-2a-Env on Gag- and Env-specific CD8^+^ T cell responses. (A) 293T cells were transiently transfected with 3 µg of the indicated plasmid DNA constructs. Following 48 h incubation, TCA-precipitated supernatants and cell lysates were separated by SDS-PAGE and analyzed by immunoblotting using p24-specific (Gag) mouse mAb (CB-4/1), and gp120-specific mouse mAb (MH23), as indicated. Molecular weight markers and positions of the detected proteins are indicated. mock: non-transfected 293T cells. (B) 293T cells were transiently transfected with equimolar amounts of the indicated plasmid DNA constructs. At 48 h post transfection, cells were permeabilized, stained with anti-p24-PE and/or anti-gp120-ALEXA 488 and analyzed by flow cytometry. (C) BALB/c mice (n = 6 per group) were inoculated i.m. with (i) an equimolar mixture of ^M^Gag and gp120 in both legs, (ii) ^M^Gag in the left, and gp120 in the right leg, or (iii) ^M^Gag-2a-gp120. After 12 days, spleen cells were isolated and tested for specific cellular immune responses by measuring IFNγ production after stimulation with Gag (black) and Env (hatched) specific peptides as above. IFNγ production was determined using FACS analysis after intracellular staining of IFNγ. Cell culture medium served as negative control. Data shown are representative of two experiments.

To clarify whether strict co-expression of Gag and gp120 in the same cell can enhance the level of suppression of Gag-specific CD8^+^ T cell responses, we first compared the expression of Gag and gp120 in vitro using (i) ^M^Gag or gp120 separately, (ii) as mixture, or (iii) both genes linked in-frame via a TaV2a sequence on one plasmid for transfecting 293T cells. Initial Western blot quality controls confirmed that Gag and Env encoded on one plasmid (^M^Gag-2a-gp120) resulted in Gag and Env expression levels comparable to the amounts seen if the plasmids encoding only one antigen were transfected either separately or as a 1∶1 equimolar mixture (Fig. 7A).

As demonstrated by FACS analyses, transfection of a mixture of ^M^Gag and gp120-encoding plasmids in 293T cells resulted in 27.1% Gag positive and 10.1% gp120 positive cells, but only 8.3% of cells were positive for both proteins (Fig. 7B). In contrast, with ^M^Gag-2a-gp120 nearly 27% of all cells expressed both antigens, whereas only 10% exclusively expressed Gag and only 0.3% exclusively gp120.

Despite these in vitro differences, Gag-specific T cell responses were strongly reduced to comparable levels both after administration of a mixture of ^M^Gag and gp120 and after the inoculation of ^M^Gag-2a-gp120. As before (Fig. 5), the spatial separation of ^M^Gag and gp120 in the left and right leg avoids the suppressive effect of gp120 on the number of of Gag-specific CD8^+^ T cells (Fig. 7C).

Thus, assuming that only a fraction of successfully transduced cells expresses both Gag and Env, direct competition for expression or a direct interaction of Gag and gp120 in the expressing cell seems unlikely to be responsible for the negative effect of Env on the induction of Gag-specific T cell responses. However, the physical proximity of both antigens, e.g. in the muscle or in the draining lymph node, with an Env, that can be secreted from transduced cells, seems to be crucial for the observed suppression of Gag-specific T cell responses.

### Deletion of the BALB/c specific epitope of gp120 prevents the reduction of Gag-specific T cells after co-administration

One study [22] already demonstrated that HIV Env interferes with the presentation of a CMV-specific CTL epitope at the level of MHC class I mediated presentation of antigenic epitopes.

**Figure 8:**
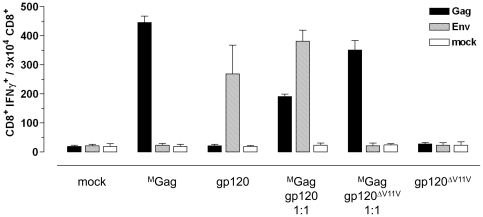
Impact of deleting the BALB/c immunodominant gp120 epitope V11V on Gag-specific CD8^+^ T cell immune responses. BALB/c mice (n = 6 per group) were i.m. inoculated with (i) equimolar amounts of the indicated plasmid DNA construct (57 µg of ^M^Gag, gp120, gp120^ΔV11V^), (ii) an equimolar mixture of ^M^Gag and gp120, or (iii) an equimolar mixture of ^M^Gag and gp120^ΔV11V^. After 12 days, spleen cells were isolated and tested for specific cellular immune responses by measuring IFNγ production after stimulation with Gag (black) or Env (hatched) specific peptides. IFNγ production was determined using FACS analysis after intracellular staining of IFNγ. Cell culture medium served as negative control. Data shown are representative of two experiments.

To evaluate a potential interference between Env and Gag at the level of peptide processing and presentation, the only known H-2^d^ restricted epitope (V11V) within the clade C CN54 envelope protein [13] was deleted, resulting in the gp120^ΔV11V^ construct (Fig. 1). Subsequently, BALB/c mice (n = 6 per group) were immunized with (i) equimolar amounts of ^M^Gag, gp120, or gp120^ΔV11V^ plasmid DNA constructs, (ii) an equimolar mixture of ^M^Gag and gp120, or (iii) an equimolar mixture of ^M^Gag and gp120^ΔV11V^ (Fig. 8). As expected, co-administration of ^M^Gag and gp120 reduced the numbers of Gag-specific T cells by about a factor of 2.3 (446±22 to 190±9), but did not significantly alter the number of induced Env-specific T cells. In contrast, levels of induced Gag-specific T cell responses following application of a mixture of ^M^Gag and gp120^ΔV11V^ were fairly high compared to the group receiving ^M^Gag alone.

Thus, deleting the gp120-specific V11V epitope from the gp120 reading frame preserves the Gag-specific T cell responses following co-administration of ^M^Gag and gp120^ΔV11V^. This suggests that competition between the Gag and gp120 antigen is most probably a result of peptide competition of the Gag-specific epitope A9I and the gp120-specific V11V for H-2^d^ restricted MHC class I epitope presentation.

## Discussion

As we have shown recently, application of our first generation HIV vaccine candidates DNA-C followed by booster immunizations with NYVAC-C, induced strong, broad and polyfunctional T cell responses in pre-clinical [13], [16] as well as clinical studies [10], [11], [12]. Unexpectedly, the induced T cell responses were predominantly directed against Env, whereas Gag-, Pol- and Nef-specific responses were less frequent and of lower magnitude.

Here, we set out to resolve this by modifying (i) the encoded antigens, (ii) the formulation, and (iii) immunization regimens to allow induction of improved Gag-, Pol- and Nef-specific T cell responses without affecting Env-specific reactivity.

Restoring the N-terminal Gag-myristoylation signal did not alter protein expression levels significantly, nor did it support formation and release of GPN particles due to the nature of this 160 kDa read-through protein. Nevertheless, this modification enhanced Gag- (∼2-fold) and Pol- (∼3-fold) specific T cell responses suggesting that alteration of intracellular trafficking, folding and/or interaction of the artificial GPN polyprotein precursor resulting from the N-terminal modification might positively impact processing of class I-restricted T cell epitopes [23].

In comparison to ^ΔM^GPN, re-introduction of the natural ribosomal frameshift, resulting in a postulated 10∶1 to 20∶1 expression ratio of Gag∶GPN [21], increased the level of Pr55 Gag expression, which translated into an increased number of Gag-specific T cells (∼6-fold). The level of Pol-specific T cell responses was only marginally reduced, which can be attributed to an overall higher protein expression compensating for the relatively lower Pol amounts compared to Gag.

The strongest Gag-specific T cell responses were observed following restoration of the budding competence, as well as enabling ribosomal frameshifting, which results in the release of VLP consisting of 90–95% Gag (∼2000 Gag molecules) and 5–10% GagPolNef (^M^Gag^FS^PN) [24], [25]. In this case, T cell responses were enhanced by a factor of 10 or more compared to the parental 160 kDa GPN. These data also nicely correlate with the results obtained after applying a 20∶1 molar mixture of Gag and PN on separated plasmids. An equimolar mixture of the latter constructs resulted in more balanced Gag- and Pol-specific T cell responses. Taken together, we were able to substantially increase Gag and PolNef protein expression levels in vitro and Gag- and Pol-specific T cell responses in vivo by (i) restoring the budding competence of Gag, (ii) re-introducing the natural viral frameshift or (iii) delivering Gag and PolNef via separate plasmids.

It is widely accepted that the combination of several antigens covering a wide spectrum of epitopes is the most promising strategy to induce broad immune responses directed against HIV. However, we observed in both humans and non-human primates [10], [16] a bias towards strong Env- and weak Gag-specific T cell responses upon co-administration of Gag and Env immunogens via viral and non-viral vectors. A second objective of this study was therefore to reveal a potential negative interference between Gag- and Env-specific immune responses, and to elaborate strategies allowing the induction of more balanced T cell responses.

First we compared antigen-specific CD8^+^ T cell responses induced after administering ^M^Gag^FS^PN or ^M^Gag with those detected after applying an equimolar mixture with gp120 encoding plasmid DNA. Co-administration of G/PN DNA mixed with the Env DNA resulted in a 2 to 10-fold decrease in Gag- and Pol-specific T cell responses, depending on the exact nature of the analyzed Gag, PN or GPN construct. Accordingly, the negative impact of Env on the stimulation of Gag- and Pol-specific T cell responses could be overcome by reducing the levels of Env encoding plasmid in the DNA vaccine mixture, thus altering the molar ratios of Env vs. Gag and PN. Furthermore, the negative effects on Gag-related CTL responses could be completely overcome by temporal or spatial separation of gp120 and Gag encoding plasmid DNAs.

Mechanisms underlying the observed Env-mediated suppression of T cell responses against other antigens are controversially discussed, and depending on the experimental design range from competition for expression [26], suppression of expression via e.g. type 1 IFN [27], Env-mediated suppression of DC activation and maturation [28], to epitope competition [22].

Along these lines, Hovav et al. [27] recently reported suppression of ovalbumin (Ova) or luciferase (Luc) expression in vivo when co-administered with an Env expression plasmid. This was accompanied by significantly reduced Ova- or Luc-specific cellular immune responses, whereby gp120-specific responses remained unaffected. Clearly in contrast to our own results as well as earlier findings by Toapanta et al. [26], this negative gp120 driven effect could not be overcome by injecting the different constructs into separate hind legs. The authors speculated that a soluble factor such as type 1 IFN, known to be produced by plasmacytoid DC triggered by gp120 [29], and to down-regulate the activity of the cytomegalovirus promoter they utilized [30], might account for the observed phenomenon.

An earlier publication reported L^d^-restricted competition between co-expressed gp160 and CMV pp89 at the level of MHC-peptide assembly [22]. Here, we could clearly show that administering an equimolar ^M^Gag/Env plasmid mixture, or one plasmid physically linking Gag and Env expression resulted in a comparable reduction of Gag-specific T cell responses, even though our in vitro analyses reveal that co-immunization of ^M^Gag and Env plasmids results in a substantial proportion of cells expressing exclusively Gag or Env (Fig. 7). Although the in vivo situation could differ from the data generated in vitro, these results also indicate that the expression of both antigens in one cell, e.g. a muscle cell, is not a stringent prerequisite for their interference.

Interestingly, deletion of the only known H-2^d^ gp120 epitope V11V within the CN54 Env abolished the negative impact of gp120 on the induction of Gag-specific T cell responses, returning levels of Gag-specific T cells back to those seen in mice receiving the ^M^Gag plasmid only (Fig. 8). Therefore, it seems highly improbable that Env either directly [26] or indirectly [27], e.g. via type 1 IFNs, negatively interferes with Gag expression, thus suppressing induction of Gag-specific T cells. Furthermore, our data also seem to exclude the possibility that an inhibitory effect of Env on DC maturation and activation causes the observed suppression of Gag-specific T cells [28].

Rather, the results presented here suggest that the Gag and Env epitopes compete for H-2^d^ MHC class I presentation. Our in vitro data strongly support the hypothesis that gp120 can be efficiently secreted from successfully transduced cells, and can therefore - in a distinct lymphatic compartment - end up together with Gag or GPN in the same antigen presenting cell, where MHC competition might happen. This idea is strongly supported by the observation that temporal or spatial separation can resolve the observed negative interference.

In conclusion, our data demonstrate that the biochemical properties of an artificial polyprotein clearly influence the levels of antigen-specific T cells, and variations in administered formulation and schedule can overcome competition for the induction of antigen-specific T cell responses. The results of this study have guided the design of ongoing pre-clinical and clinical trials.
